# Combined 23-gauge transconjunctival vitrectomy and scleral fixation of intraocular lens without conjunctival dissection in managing lens complications

**DOI:** 10.1186/s12886-018-0776-4

**Published:** 2018-04-23

**Authors:** Ling Yeung, Nan-Kai Wang, Wei-Chi Wu, Kuan-Jen Chen

**Affiliations:** 1 Department of Ophthalmology, Chang Gung Memorial Hospital, Keelung, Taiwan; 2 College of Medicine, Chang Gung University, Taoyuan, Taiwan; 3 Department of Ophthalmology, Chang Gung Memorial Hospital, No 5, Fu Hsing Street, Kuei Shan, Taoyuan, Taiwan

**Keywords:** Cataract surgery, Intraocular lens, Lens dislocation, Lens subluxation, Scleral fixation

## Abstract

**Background:**

To evaluate the safety and efficacy of combined 23-gauge transconjunctival pars plana vitrectomy and scleral fixation of intraocular lens (IOL) without conjunctival dissection.

**Methods:**

A retrospective study in Chang Gung Memorial Hospital, Keelung and Taoyuan, Taiwan. Patients receiving combined 23-gauge transconjunctival pars plana vitrectomy and scleral fixation of IOL without conjunctival dissection were enrolled. The ocular findings, causes of lens complication, surgical procedures, type of IOL used, and complications were documented.

**Results:**

We included 40 eyes from 39 patients (27 male, 12 female) with a mean age of 59.5 [standard deviation (±) 14.8] years old. The mean follow-up duration was 6.8 ± 5.4 months. The cause of lens complications was ocular trauma in 24 (60%) eyes, cataract surgery complications in 11 (28%) eyes, and spontaneous subluxation of crystalline lens in 5 (13%) eyes. The overall best corrected visual acuity (BCVA) (logMAR) improved from 1.359 ± 0.735 to 0.514 ± 0.582 (*p* < 0.001). The BCVA also improved significantly in each group with different causes of lens complications. Preoperative BCVA was the only factor associated with the postoperative visual outcome (*p* = 0.008). Most surgery-related complications were self-limited, including mild vitreous hemorrhage (5%), microhyphema (5%), transient elevated intraocular pressure (3%), and transient hypotony (3%). Cystoid macular edema and IOL decentration was found in 3 (8%) eyes and 1 (3%) eye respectively.

**Conclusions:**

Combined 23-gauge transconjunctival vitrectomy and scleral fixation of IOL without conjunctival dissection is effective and safe in managing a wide variety of lens complications, with good postoperative comfort and visual recovery.

**Trial registration:**

Retrospective study, not applicable.

**Electronic supplementary material:**

The online version of this article (10.1186/s12886-018-0776-4) contains supplementary material, which is available to authorized users.

## Background

Lens-related complications are common in clinical practice. These include crystalline lens subluxation or dislocation due to ocular trauma or systemic diseases (such as Marfan syndrome), complicated cataract surgeries with retained lens materials or inadequate capsular support for intraocular lens (IOL), and spontaneous IOL dislocation. More devastating and serious complications such as persistent corneal edema, secondary glaucoma, uveitis, vitreous hemorrhage, cystoid macular edema and retinal detachment may occur if untreated [1]. Vitrectomy with or without lensectomy are the standard procedures to clean up the vitreous incarceration and lens materials, followed by IOL implantation, reposition or exchange [1, 2]. Nowadays, small gauge (23-gauge or 25-gauge) transconjunctival pars plana vitrectomies have the advantages of avoiding conjunctival dissection, smaller sclerotomies, and omitting sutures required for sclerotomy and conjunctival wounds when compared with 20-gauge vitrectomies. This improves the postoperative comfort in patients.

After vitrectomy, IOL can be implanted either into the anterior chamber (AC) or the posterior chamber (PC). Although it involves a more complicated surgical process, PC IOL in ciliary sulcus is more compatible with the physiological location of the lens and less likely to damage the angle structure and corneal endothelial cell. Hoffmann et al. proposed a method to fixate the in-the-bag IOL dislocation by creating a “reverse” scleral pocket [3]. The “reverse” scleral pocket was dissected outward from a clear cornea incision and conjunctival dissection was not required. This method was also reported in the handling of other IOL complications [4, 5]. By combining this technique with the small gauge transconjunctival pars plana vitrectomy, a wide variety of lens complications can theoretically be managed by a single surgery with a more comfortable postoperative condition and faster visual recovery. However, the efficacy and safety of these concurrent surgeries are unknown.

The purpose of the current study is to evaluate the safety and efficacy of concurrent 23-gauge transconjunctival pars plana vitrectomy and scleral fixation of IOL by a modified version of Hoffmann’s technique. No conjunctival dissection was needed throughout the whole surgery. The postoperative complications, choice of IOLs, and the surgical tips are also discussed.

## Methods

This was a retrospective study conducted at Chang Gung Memorial Hospital, Keelung and Taoyuan, Taiwan. The study was approved by the Institutional Review Board of Chang Gung Memorial Hospital and adhered to the tenets and guidelines of the Declaration of Helsinki. Patients receiving concurrent 23-gauge transconjunctival pars plana vitrectomy and scleral fixation of IOL without conjunctival dissection between October 2014 and December 2015 were included. The major exclusion criteria were: (1) using 20-gauge pars plana vitrectomy; (2) performing scleral fixation of IOL without simultaneous pars plana vitrectomy; (3) performing scleral fixation with other surgical techniques different from this study; (4) postoperative follow up of less than 1 month. Medical records, including clinical history, cause of lens complication, findings in ocular examinations, surgical procedures, type of IOL and postoperative complications were reviewed. The postoperative best corrected visual acuity (BCVA) was defined as the best visual acuity measurement between 1 to 3 months after surgery. The BCVA was measured using the Snellen chart and was converted to a logarithm of the minimum angle of resolution (logMAR) visual acuity for calculation. Counting fingers, hand movement, and light perception vision were allocated to 1/200, 1/400 and 1/800 respectively [6].

### Surgical techniques

(Also see Additional file 1: Video S1 which demonstrates the surgical techniques)


**Additional file 1:** Video S1. Description of data: Demonstrates the surgical techniques in this manuscript. (MP4 38,911 kb)


**Fig. 1:**
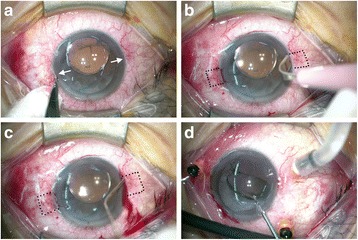
**a** A #11 beaver blade was used to create 2 corneal incisions (white arrows) just anterior to the conjunctival insertion at 180 degrees apart on the desired meridian. **b** A metal crescent blade was used to make 2 scleral pockets by posterior lamellar dissection from the corneal incisions to about 3 mm posterior to the incisions. **c** A spatula was used to check the width and depth of the scleral pockets. The margins of the scleral pockets were indicated by the black dots in (**b**) and (**c**). **d** Standard 3-port 23-gauge transconjunctival pars plana vitrectomy trocars were set up with infusion at the temporal inferior quadrant. 23-gauge micro forceps were used to grasp the intraocular lens from ciliary sulcus into the anterior chamber

**Step 1: Creating Hoffmann scleral pockets.** This technique is modified from that described by Hoffmann et al. [3]. A #11 beaver blade was used to create 2 corneal incisions just anterior to the conjunctival insertion at 180 degrees apart from the desired meridian (Fig. 1a). The incisions were about 1 clock hour in width and one-third of the corneal thickness in depth. Some surgeons may omit this procedure to avoid surgically induced astigmatism (Additional file 1: Video S1). A metal crescent blade (Crescent Knife, Satin Cresecent™ Angled Bevel Up, Alcon) was used to make 2 scleral pockets by posterior lamellar dissection from the corneal incisions to about 3 mm posterior to the incisions (Fig. 1b). A spatula was used to check the width and depth of the scleral pockets (Fig. 1c).

**Step 2: Setting 23-gauge transconjunctival pars plana vitrectomy system.** Standard 3-port 23-gauge transconjunctival pars plana vitrectomy trocars were inserted at 3.5 - 4 mm from the limbus with infusion at the inferotemporal quadrant (Fig. 1d). Pars plana vitrectomy, pars plana lensectomy or other vitreoretinal surgical procedures can be done at this stage; or alternatively, at a later stage after scleral fixation of IOL. A clear corneal incision was done at the superior quadrant by a 2.65 mm blade (EdgeAhead® Slit Knife 2.65 mm(40°), Beaver-Visitec International Ltd., Warwickshire, UK) The anterior chamber was filled with viscoelastic materials. In patients who required IOL reposition, the original IOL was moved to the anterior chamber by a 23-gauge micro forceps (Fig. 1d). In crystalline lens subluxation patients with some residue zonules and intact capsular bags, we could hold the capsular bag by iris retractors, and performed phacoemulsification to remove the cataract as much as possible if there was no vitreous prolapsed.

**Fig. 2:**
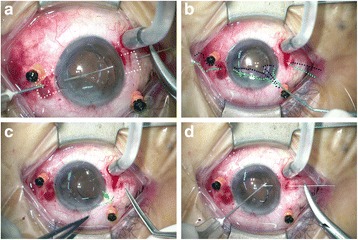
**a** A single-armed 10-0 Polypropylene suture on a long straight needle was inserted into eye at one side at 1.5 mm posterior to the surgical limbus (right side). A 27-gauge needle was also inserted into the eye at the scleral pocket at 1.5 mm posterior to the surgical limbus in the opposite side (left side). The long straight needle was docked with the 27-gauge needle and externalized. Then this procedure was repeated in the opposite direction. The margins of the scleral pockets were indicated by white dots. **b** The two 10-0 polypropylene sutures (indicated by blue dots and green dots) were pulled out from the superior corneal incision by a Jaffe-Knolle Iris Hook. **c** One haptic of the IOL was pulled out from the superior corneal incision and was tightened with the 10-0 polypropylene sutures (green arrow). The haptic was then pushed back into the anterior chamber and the process repeated with another haptic. **d** Alternatively, we can also pass the 10-0 polypropylene suture first beneath the haptic and then above the haptic to form a loop to fix the haptic to ciliary sulcus

**Step 3: Fixating the IOL haptics with 10-0 polypropylene sutures.** A single-armed 10-0 polypropylene suture on a long straight needle (AUM-5 & SC-5, Alcon) was inserted into the eye at one side by passing through the conjunctiva and the full thickness of the scleral pocket at 1.5 mm posterior to the surgical limbus (Fig. 2a). A 27-gauge needle was passed through the conjunctiva and scleral pocket at 1.5 mm posterior to the surgical limbus in the opposite side. The long straight needle was docked with the 27-gauge needle and externalized (Fig. 2a). The above procedures were repeated in the opposite direction. The long straight needle was inserted again into the eye at the location just beside the exit. It was docked with a 27-gauge needle again and passed out beside the initial entry site. The 10-0 polypropylene sutures were pulled out from the superior corneal incision by a Jaffe-Knolle Iris Hook (Fig. 2b). Each 10-0 polypropylene suture was cut at the center. In patients who required IOL implantation, a foldable IOL was inserted into the anterior chamber at the current stage. One haptic of the IOL will be pulled out from the superior corneal incision and tightened with the two 10-0 polypropylene sutures from one of the scleral pockets (Fig. 2c). The haptic was then pushed back into the anterior chamber; and the process repeated with another haptic and the two 10-0 polypropylene sutures from another scleral pocket. Alternatively, for those IOLs with haptics that are difficult to externalize, we can pass the 10-0 Polypropylene suture first beneath the haptic and then above the haptic to form a loop to fix the haptic to ciliary sulcus (Fig. 2d).

**Fig. 3:**
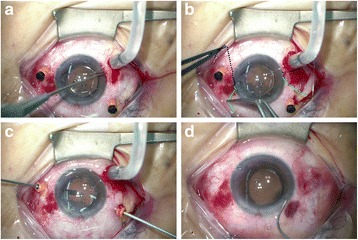
**a** The 10-0 polypropylene sutures were retrieved out from scleral pockets by a Sinskey hook. The IOL centricity was ensured by adjusting the tightness of sutures in both scleral pockets. **b** The sutures buried themselves inside the scleral pocket spontaneously when they were tightened. **c** The residue vitreous, lens materials, or viscoelastic materials were then removed. **d** The 23-gauge vitrectomy trocars were removed. The corneal wound was hydrated by balanced salt solution or sutured with 10-0 nylon if necessary

**Step 4: Retrieved the 10-0 polypropylene sutures from scleral pockets and tightened the knots.** The sutures were retrieved from the scleral pockets by a Sinskey hook (Fig. 3a). The IOL was moved to the posterior chamber and the centration was ensured by adjusting the tightness of sutures in both scleral pockets (Fig. 3b). The knots buried themselves inside the scleral pocket spontaneously when they were tightened.

**Step 5: Cleaning up and concluding the surgery.** Residue vitreous, lens materials, or viscoelastic materials were all removed by vitrectomy or simcoe cannula (Fig. 3c). The 23-gauge vitrectomy trocars were then removed. The corneal wound was hydrated by balanced salt solution or sutured with 10-0 nylon if necessary (Fig. 3d).

### Statistical analysis

Statistical analysis was performed using SPSS 17.0 (SPSS Inc., Chicago, IL). Continuous data were reported as the mean ± standard deviation, and categorical data were reported as n (%).Paired sample *t*-test and chi-square test were used for comparing the preoperative BCVA to the postoperative BCVA. One way ANOVA was used to determine the difference in the preoperative BCVA and the postoperative BCVA among eyes with different causes of lens complications. Linear regression model was used to evaluate the factors associated with the postoperative BCVA. A *p* value < 0.05 was considered statistically significant in this study.

## Results

A total of 40 eyes from 39 patients (male: female = 27:12) were enrolled in this study. The mean age was 59.5 [range 22 - 90, standard deviation (±) 14.8] years old and the mean postoperative follow-up duration was 6.8 (± 5.4) months. Table 1 shows the cause of lens complications, prior surgeries and the preoperative lens status. The most common cause of lens complications was ocular trauma (24 eyes, 60%), which included open globe injury in 6 eyes (15%) and closed globe injury in 18 eyes (45%), followed by cataract surgery complications (11 eyes, 28%) and spontaneous subluxation of crystalline lens (5 eyes, 13%). The surgical procedures and type of IOL used in the current surgery are summarized in Table 2. All patients received 23-gauge vitrectomy and scleral fixation of IOL. Phacoemulsification or pars plana lensectomy was performed in 17 (43%) eyes. Foldable (soft) IOLs were implanted in 31 (78%) eyes and PMMA IOLs were used in 4 (10%) eyes.

**Table 1:** Clinical characteristics of patients

Total number of eyes	40
Total number of patients	39
Male: Female	27: 12
Age, mean ± standard deviation	59.5 ± 14.8
Cause of lens complications, *n* (%)	
Ocular trauma	24 (60)
Cataract surgery complications	11 (28)
Spontaneous subluxation of crystalline lens	5 (13)
Prior surgeries, *n* (%)	
Cataract surgery	19 (48)
Vitrectomy	4 (10)
Primary repair of open globe injury	2 (5)
Scleral buckle	1 (3)
Scleral fixation	1 (3)
Preoperative lens status, *n* (%)	
Crystalline lens subluxation / dislocation	19 (48)
IOL dislocation	13 (33)
Aphakia	6 (15)
Retained lens fragments	2 (5)

*Abbreviation: IOL* intraocular lens

**Table 2:** Surgical procedures and type of intraocular lens used in the study

	Number of eyes (%)
Procedures performed	
23-gauge pars plana vitrectomy	40 (100)
Phacoemulsification / pars plana lensectomy	19 (48)
Epiretinal membrane peeling	1 (3)
IOL related procedures	
IOL implantation	28 (70)
IOL reposition	10 (25)
IOL exchange	2 (5)
Type of IOL used	
Rayner 570C / 620H / 920H / 970C	20 (50)
Alcon SA60AT / SN60WF	6 (15)
AMO Sensar AR40e	3 (8)
AMO AAB00	1 (3)
Bausch & Lomb MX60	1 (3)
Alcon CZ70BD	4 (10)
Using original IOL which type unknown	5 (13)

*Abbreviation: IOL* intraocular lens

Table 3 shows the changes in BCVA. None of the patients showed decreased BCVA after the surgery. The BCVA improved significantly from 1.359 (Snellen equivalent 20/457) preoperatively to 0.514 (Snellen equivalent 20/65) postoperatively (*p* < 0.001) in study patients as a whole. The BCVA improved by 3 lines or more in 32 (80%) eyes. When the patients were divided into three groups according to different causes of lens complications (i.e. ocular trauma, cataract surgery complications, and spontaneous subluxation of crystalline lens), there were no intergroup differences in the preoperative BCVA and the postoperative BCVA. All three groups of patients showed significant improvement in BCVA after the surgery. (Table 3) The linear regression model showed that only the preoperative BCVA (*p* = 0.008) was correlated with the postoperative BCVA. Other factors including age, gender, cause of lens complication, prior cataract surgery, prior vitrectomy, concurrent phacoemulsification / lensectomy, type of IOL procedure, and type of IOL used were not correlated with the postoperative BCVA.

**Table 3:** Change in the best corrected visual acuity

	Preoperative	Postoperative	*p* value
BCVA level, *n* (%)			0.001*
< 20/200	19 (48)	3 (8)	
20/200 - 20/40	21 (53)	22 (55)	
> 20/40	–	15 (38)	
BCVA in logMAR (Mean ± SD)			
Overall (*n* = 40)	1.359 ± 0.735	0.514 ± 0.582	< 0.001 †
Ocular trauma (*n* = 24)	1.265 ± 0.669	0.477 ± 0.603	< 0.001 †
Cataract surgery complications (*n* = 11)	1.675 ± 0.866	0.711 ± 0.609	0.006 †
Spontaneous subluxation of crystalline lens (*n* = 5)	1.114 ± 0.645	0.264 ± 0.295	0.036 †
*p* value	0.230‡	0.328‡	

*Abbreviation: BCVA* best corrected visual acuity, *logMAR* logarithm of the minimum angle of resolution, *SD* standard deviation

*Comparing the postoperative BCVA level to the preoperative BCVA level, *p* value was calculated by chi-square test

† Comparing the postoperative BCVA (logMAR) to the preoperative BCVA (logMAR), *p* values were calculated by paired sample t-test

‡ Comparing the BCVA among 3 different causes of lens complications (i.e. ocular trauma, cataract surgery complications, and spontaneous subluxation of crystalline lens), *p* values were calculated by one way ANOVA

No major intraoperative complications such as retinal tear, retinal detachment, or suprachoroidal hemorrhage were noted. The postoperative complications are summarized in Table 4. The majority of surgery-related complications were self-limited, such as mild vitreous hemorrhage, microhyphema, transient elevated intraocular pressure, and transient ocular hypotony. All of these self-limited complications resolved within 1 to 2 weeks after surgery. Three (8%) eyes with cystoids macular edema required topical nonsteroidal anti-inflammatory (NSAID) drugs or intravitreal injection of steroid. One (3%) eye receiving IOL reposition had IOL decentration at 2 months after surgery due to vigorous eye rubbing.

**Table 4:** Postoperative complications

	Number of eyes (%)
Complications related to prior underlying ocular disorders	
Traumatic optic neuropathy	2 (5)
Recurrent retinal detachment	1 (3)
Corneal irregular astigmatism	1 (3)
Progression of epiretinal membrane	1 (3)
Complications related to current surgical procedures	
Cystoid macular edema	3 (8)
Mild vitreous hemorrhage	2 (5)
Mild hyphema	2 (5)
Transient elevated intraocular pressure	1 (3)
Transient hypotony	1 (3)
Intraocular lens decentration	1 (3)
Intraocular lens dislocation	0 (0)

## Discussion

Our results show that combined 23-gauge transconjunctival vitrectomy and scleral fixation of IOL without conjunctival dissection is an effective and safe method in handling a wide variety of lens complications within a single surgery. Preoperative BCVA is the only factor correlated with postoperative visual outcome. Concurrent phacoemulsification / lensectomy, type of IOL procedure (implantation / reposition / exchange), and type of IOL used were not associated with visual outcome. Most complications related to the current surgical procedure were self-limited.

**Fig. 4:**
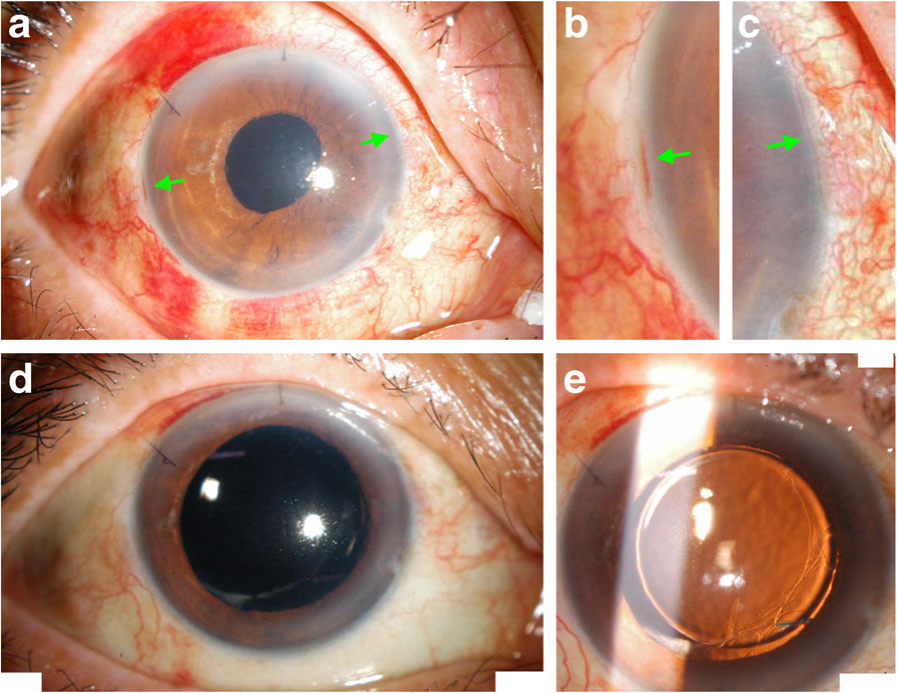
External photos of the same patient in Figs. 1, 2 and 3. **a** Minimal residual corneal edema and conjunctival hemorrhage at 3 days after surgery. Incison wounds (indicated by green arrows) of both scleral pockets were healing well. **b-c** The manified image of temporal and nasal scleral pocket incision wounds (green arrows). **d** No more corneal edema and conjunctival hemorrhage could be found at 10 days after surgery. **e** Intraocular lens centration was good

In patients without adequate capsular support, many surgical methods were introduced to fixate the PC IOL into the ciliary sulcus. However, most of them involve conjunctival peritomy and creating a scleral flap or glove to embed the stitches or haptics of IOL [2]. In this study, by combining a modified form of Hoffmann’s “reverse” scleral pocket technique and 23-gauge transconjunctival vitrectomy, a wide variety of lens complications were treated without conjunctival periotomy. These provided rapid surgical wounds recovery and less postoperative discomfort in patients (Fig. 4).

No major intraoperative complications were noted. Most of the vision-threatening complications were related to prior underlying ocular diseases (Table 3). Most complications related to current surgical procedures were self-limited, including mild vitreous hemorrhage, microhyphema, transient elevated intraocular pressure, and transient hypotony. All of these complications resolved within 1 to 2 weeks. Cystoid macular edema occurred in 8% of patients. This is comparable to previous studies [7]. Pseudophakic cystoid macular edema was observed on optical coherence tomography in 4-11% of patients, and may be further increased in complicated cataract surgeries [7]. In considering the complexity of the surgeries in this study, it is reasonable that 8% of eyes developed cystoid macular edema. Nowadays, most cystoid macular edema can be successfully treated by topical NSAID or local (intravitreal or sub-Tenon) injection of corticosteroid [7]. In this study, IOL decentration was noted in an 86 years old male patient after vigorous eye rubbing. None of the patients had IOL dislocation to vitreous cavity after surgery.

In our study, a soft foldable IOL was used in 31 (78%) eyes. Recent reports showed that different types of foldable IOLs can be used safely for scleral fixation in aphakic eyes [8–12]. The advantages include small incision, short surgical time, less likelihood of intraoperative hypotony-related complications, and faster recovery [8–12]. For patients with IOL subluxation / dislocation, we prefer to use the original IOLs for reposition whenever possible. The advantage of this is that only a small corneal incision (2.2 mm - 2.65 mm) is required for externalization and fixation of the haptics of the IOL. The small corneal incision can be easily sealed by balanced salt solution hydration or a 10-0 nylon suture at the end of surgery. For patients with crystalline lens subluxation / dislocation or in aphakic status, we prefer to use closed-loop foldable IOLs (e.g. Rayner 570C / 620H / 920H / 970C). In fact, this is the most common type of IOL used in this study. It is easy for the suture to pass through the closed-loop and we do not need to worry about the suture slipping out of the haptic after surgery. A 2.65 mm – 2.8 mm corneal incision is adequate for the implantation of a foldable IOL. Other factors that we may consider include the presence of corneal astigmatism, the size of iris defect, and the availability of specific types of IOL among the participating hospitals. However, our study showed that the current surgical technique is feasible for a wide variety of IOLs. The suture technique is quite similar for different type of IOLs. The type of IOL used was not correlated with postoperative visual outcome.

The surgical technique in this study is moderately difficult in its learning curve. The most difficult part of the surgery is in creating the scleral pockets. There are some tips in this procedure. Firstly, it is better to create the scleral pockets at the beginning of the surgery, before inserting 23-gauge vitrectomy trocars and performing anterior chamber paracentesis. This maintains adequate intraocular pressure and firmness of eyeball for easier dissection of the scleral pocket. Secondly, the dissection of the scleral pockets may be difficult at some surgical angles. This can be facilitated by rotating the eyeball to the opposite direction, so the scleral pocket dissection can be done at the horizontal plane in an easier manner. Thirdly, we recommend checking the width and depth of the scleral pocket with a spatula after dissection (Fig. 1c). The depth of the scleral pocket can be easily estimated by measuring the bloodstain or water mark on the spatula. Inadequate width and depth of the scleral pockets may lead to difficulty in retrieving the sutures in Step 3.

Our study was limited by its retrospective nature and a short follow up duration. The follow up duration is relative short in this study because most referred patients will be referred back to primary care general ophthalmologists once their postoperative conditions have stabilized. However, our short-term results are promising. Our study also limited by lacking a comparative group. It requires further studies to evaluate the success rate, the visual outcome, and the complications when compare to other surgical methods.

## Conclusions

Combined 23-gauge transconjunctival vitrectomy and scleral fixation of intraocular lens without conjunctival dissection is an effective and safe surgical technique in handling a wide variety of lens complications within a single surgery. Visual acuity improved significantly in all groups with different causes of lens complications. Most complications related to the current surgical technique are self-limited.

## Abbreviations

AC: Anterior chamber
BCVA: Best corrected visual acuity
IOL: Intraocular lens
LogMAR: Logarithm of the minimum angle of resolution
NSAID: Nonsteroidal anti-inflammatory
PC: Posterior chamber
